# Breast Cancer Pathology Turnaround Time in Botswana

**DOI:** 10.1200/JGO.17.00090

**Published:** 2017-11-09

**Authors:** Yehoda M. Martei, Mohan Narasimhamurthy, Pooja Prabhakar, Jeré Hutson, Dipho I. Setlhako, Sebathu Chiyapo, Doreen Ramogola-Masire, Ignetious Makozhombwe, Michael Feldman, Mukendi K.A. Kayembe, Surbhi Grover

**Affiliations:** **Yehoda M. Martei**, **Jeré Hutson**, **Doreen Ramogola-Masire**, **Michael Feldman,** and **Surbhi Grover**, University of Pennsylvania, Philadelphia, PA; **Mohan Narasimhamurthy**, **Doreen Ramogola-Masire**, and **Mukendi K.A. Kayembe**, University of Botswana; **Dipho I. Setlhako** and **Surbhi Grover**, Princess Marina Hospital; **Sebathu Chiyapo**, Gaborone Private Hospital; **Doreen Ramogola-Masire** and **Surbhi Grover**, Botswana University of Pennsylvania Partnership; **Ignetious Makozhombwe**, Diagnofirm Medical Laboratories; **Mukendi K.A. Kayembe**, National Health Laboratory, Gaborone, Botswana; and **Pooja Prabhakar**, University of Texas Southwestern Medical Center, Dallas, TX.

## Abstract

**Purpose:**

Quality pathology is critical for timely diagnosis and management of breast cancer. Few studies have analyzed pathology turnaround time (TAT) in sub-Saharan Africa. The purpose of this study was to quantify TAT for breast cancer specimens processed by the National Health Laboratory and Diagnofirm Laboratory in Gaborone, Botswana, and additionally compare TAT before and after 2012 to evaluate the effect of pathology scale-up interventions by the Ministry of Health and Wellness.

**Methods:**

Retrospective analyses of TAT were performed for breast specimens submitted to the two laboratories from 2011 to 2015. TAT was calculated as the time from specimen collection and receipt in the laboratory to the date of final report sign-out. Descriptive statistics and rank sum test were used to compare temporal trends in TAT before and after 2012.

**Results:**

A total of 158 breast biopsy, 219 surgical, and 218 immunohistochemistry (IHC) specimens were analyzed. The median TAT in 2015 was 6 and 7 days for biopsy and IHC specimens, respectively, and 57.5 days for surgical specimens. There was a significant decrease in median TAT for biopsy specimens from 21.5 days in 2011 to 2012 compared with 8 days in 2013 to 2015 (*P* < .001). There was also a significant decrease in median TAT for IHC specimens during the same period (*P* < .001). However, there was no significant decline in median TAT for surgical specimens.

**Conclusion:**

The scale-up of pathology personnel and infrastructure by the Ministry of Health and Wellness significantly reduced median TAT for biopsy and IHC specimens. TAT for surgical specimens remains suboptimal. Efforts are currently under way to decrease TAT for surgical specimens to 7 days.

## INTRODUCTION

Breast cancer is the most common cancer affecting women worldwide, with approximately two thirds of breast cancer deaths occurring in low- and middle-income countries.^1^ In Botswana, a middle-income country in sub-Saharan Africa, breast cancer represents 18% of all cancers diagnosed and 12.5% of cancer-associated deaths among women.^2^

Histopathologic evaluation is a critical component of scaling up cancer care for patients with breast cancer. Successful management of breast cancer requires accurate, complete, and timely pathology reporting. It provides initial diagnosis and prognostic information that guides additional work-up and treatment decisions. Recent studies have demonstrated that delays in breast cancer presentation and diagnosis are partly a result of in-hospital system delays, which include the timeliness of obtaining pathology services.^3^ However, few studies from sub-Saharan Africa have quantified the pathology turnaround time (TAT) and its potential effect on delays in initiating appropriate treatment of patients with breast cancer. The Botswana Ministry of Health and Wellness (MOHw) expanded its pathology capacity in 2012 by doubling the number of pathologists from two to four. Compared with neighboring countries in the region, Botswana and South Africa have the highest ratios of pathologists to population served in sub-Saharan Africa. Botswana has approximately one pathologist per 500,000 people, which is much higher than the ratio in neighboring countries, where the pathologists-to-population ratio is in excess of 1 to 2.5 million people.^4,5^ However, this ratio is less than optimal to deliver quality timely pathology services compared with Western countries. For instance, the pathologist-to-population ratio in North America is 1 to 17,544 people.^6^

The National Health Laboratory (NHL) is the major pathology service provider available in the public health sector in Botswana. NHL provides histopathology and partial immunohistochemistry (IHC) services to nine hospitals, including primary, district, and referral hospitals located in the southern half of the country. Diagnofirm Medical Laboratories (DML) is an independent laboratory that processes specimens from the private and public sectors and is a major referral for specialized pathology services within the country. The aim of this study was to measure the pathology TAT for breast cancer specimens analyzed at the NHL and DML in Botswana between 2011 and 2015. We also examined the effect of expanding pathology services in the country on the TAT for breast cancer specimens.

## METHODS

### Laboratory Set-Up

The NHL is located in Gaborone and currently employs four qualified surgical pathologists, two cytoscreeners, and five histology laboratory technicians and scientists. Two additional pathologists and four laboratory technicians and scientists have full-time appointments within the Faculty of Medicine at the University of Botswana and assist with clinical services and the training of eight residents.

Before 2012, there were only two pathologists at the NHL. Furthermore, the entire histology work process was manual. Through the facilitation of representatives of the MOHw and National Cervical Cancer Screening Program, the American Society of Clinical Pathology (ASCP) sent a team of pathologists to Botswana to clear pathology backlog for possible cervical cancer cases.^7^ The collaboration initially started in cervical cancer but was expanded to cover other cancer cases. Given that the backlog was a result of a shortage in pathology personnel and manual processing, the ASCP recommended that the MOHw acquire automated and semiautomated tissue processing equipment and a whole slide imaging system. These were procured and installed in 2013. Currently, the NHL reviews approximately 6,500 benign and malignant biopsies and surgical specimens annually. Malignant breast cancer represents less than 5% of cases.

DML is a private laboratory service in Botswana with 12 locations throughout the country. The laboratory provides expert consultation and referrals for breast IHC cases and other specialized pathology testing services not available at the NHL. Currently, DML reviews 1,400 benign and malignant biopsies and surgical specimens annually, of which approximately 4% are malignant breast cancer.

### Data on TAT

To evaluate pathology TAT, data were abstracted retrospectively from pathology reports generated for patients with histologically confirmed breast cancer diagnoses evaluated at NHL and DML between January 2011 and December 2015. This represents the period for which complete pathology data were available electronically. Data were collected on biopsy specimens, surgical specimens, and specimens for IHC testing for estrogen receptor, progesterone receptor, and human epidermal growth factor receptor 2 status.

TAT was computed for the following components of pathology assessment by obtaining the dates of the sequential steps in specimen processing and analysis: (1) date of specimen collection, defined as the date of biopsy or surgical procedure; (2) date of receipt in the laboratory; (3) date of gross pathology, defined as the date the specimen was cut for histology in the laboratory; (4) date of gross examination, defined as the date gross examination was completed on the specimen; and (5) date of final report sign-out, defined as the date the histology report was authorized by the pathologist. If IHC was performed, the final sign-out date was automatically reassigned by the electronic medical record system to the date the IHC addendum was reported, and the original histology sign-off date was automatically overridden.

We calculated the mean and median TAT for specimen collection to final sign-out and the other components of TAT, including specimen collection to receipt in laboratory, specimen collection to gross examination completion, and specimen in laboratory to final sign-out to understand which components affect overall TAT. Not all pathology reports had complete dated information for the different steps in specimen processing and analyses. The different components of TAT were limited to specimens that had the specified processing dates. Median TAT for specimen collection to receipt in laboratory was stratified by private versus public laboratory and location of referral hospital and district to provide laboratory and regional variations for the respective TAT. This analysis was limited to the few specimens for which a referral hospital was indicated on the pathology report.

### Statistical Analysis

All data were entered into a Research Electronic Data Capture database and analyzed in STATA (STATA, College Station, TX). Rank sum test was used to test the difference between the median TAT for pathology reported in 2011 to 2012, compared with 2013 to 2015. All probabilities were two-tailed, and *P* values < .05 were regarded as statistically significant.

### Ethical Review

This study was reviewed and approved by the Institutional Review Board of the University of Botswana, the Health Research and Development Committee of MOHw, and the Institutional Review Board of DML.

## RESULTS

### Volume of Pathology Processed in NHL and DML

Data were available for 158 breast biopsy specimens, 219 surgical specimens, and 218 IHC specimens processed during the study period. This included a total of 53 specimens from DML. The number of biopsy, surgical, and IHC specimens processed between 2011 and 2015 are listed in Table 1. IHC was performed on either biopsy specimens or surgical specimens.

**Table 1:**
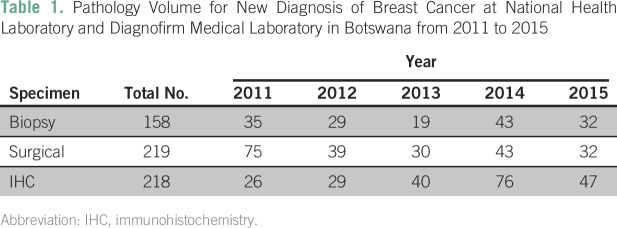
Pathology Volume for New Diagnosis of Breast Cancer at National Health Laboratory and Diagnofirm Medical Laboratory in Botswana from 2011 to 2015

### Analysis of Pathology TAT

The mean and median of the different components of the TAT of breast cancer specimens are listed in Table 2. The time from specimen collection to receipt in the laboratory was shorter than the time interval from specimen reception to report sign-out. The time from collection to gross examination was extended but did not account for all the difference observed in overall TAT from specimen collection to report sign-out. In addition, respective TAT for private laboratories was consistently shorter than the TAT for specimens processed within the public sector. We noted a range of TAT from collection to final sign-out extending up to 260 days for biopsy and 578 for surgery and IHC. These outliers are most likely a result of specimens where IHC was not reflexively ordered at the time of initial diagnosis or treatment, but requested by the treating clinician at a later date, at the time of either local or distant recurrence. If IHC is later reported on a biopsy or surgical specimen, the electronic record automatically replaces the date of any previous sign-out dates with the IHC sign-out date. We estimate that most of the prolonged outliers were a result of IHC requests on previous biopsy or surgical specimens requested in this manner.

**Table 2:**
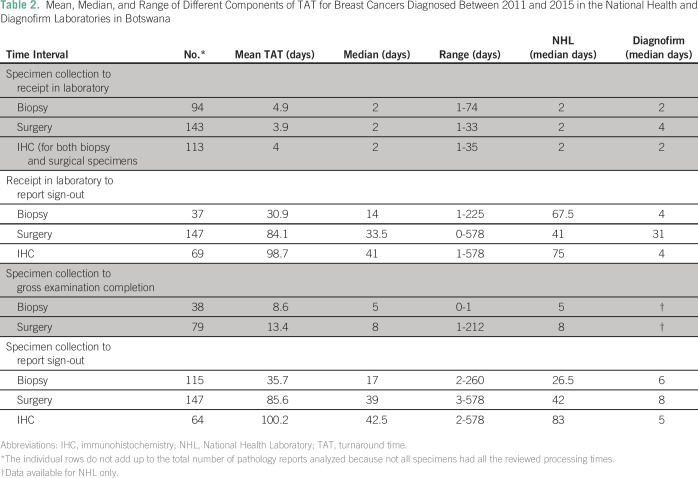
Mean, Median, and Range of Different Components of TAT for Breast Cancers Diagnosed Between 2011 and 2015 in the National Health and Diagnofirm Laboratories in Botswana

**Fig 1:**
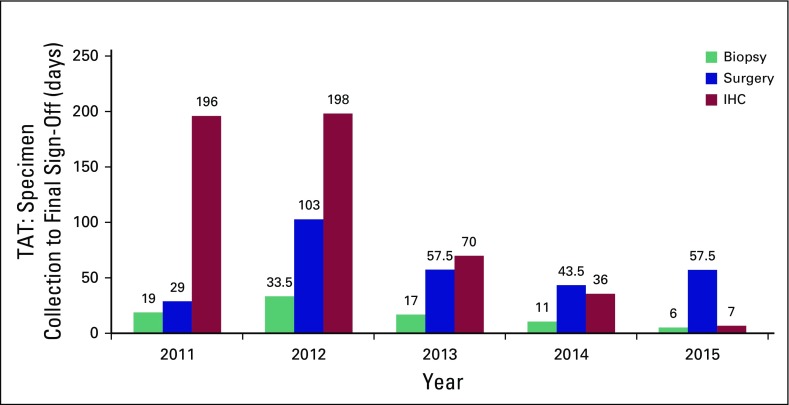
Median turnaround time (TAT) of specimen collection to report sign-out from 2011 to 2015 for new diagnosis of breast cancer. IHC, immunohistochemistry.

Figure 1 shows the median TAT trend for specimen collection to final sign-off by individual years. There was a 62.3% significant decline in median TAT from 21.5 days in 2011 to 2012 compared with 8 days in 2013 to 2015 (*P* < .001) for biopsy reports. Similarly, there was an 82% decline in TAT from 280 days in 2011 to 2012 to 50 days in 2013 to 2015 for IHC reporting (*P* < .001). There was no significant decline in TAT for surgical specimens analyzed in 2011 to 2012, compared with specimens analyzed after that period when pathology services were increased (*P* = .7123).

NHL and DML received and analyzed pathology specimens from nine major hospitals in Botswana. Table 3 lists the median TAT for facilities where the referral institution is listed and for which TAT data are available. A total of 44% (41 of 93) of biopsy specimens and 63% (90 of 142) of surgical specimens were received from Princess Marina Hospital (PMH), the major cancer referral center in the southern part of the country. Table 3 lists the median TAT data from surgery or biopsy to receipt in the laboratory for specific hospitals and geographic locations within Botswana. The data highlight a trend for shorter TAT for biopsy and surgical specimens processed from PMH compared with all other hospitals.

**Table 3:**
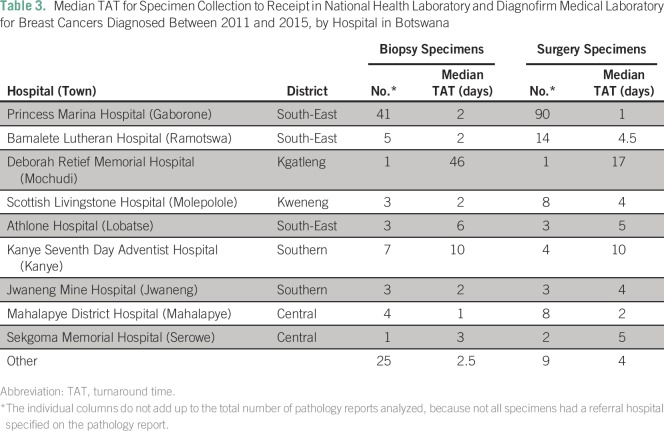
Median TAT for Specimen Collection to Receipt in National Health Laboratory and Diagnofirm Medical Laboratory for Breast Cancers Diagnosed Between 2011 and 2015, by Hospital in Botswana

## DISCUSSION

Although there are anecdotal data to suggest prolonged pathology TAT in sub-Saharan Africa, our study on breast cancer pathology TAT in Botswana is one of the few attempts in the region to quantify pathology TAT for patients with breast cancers seen in sub-Saharan Africa. In two recent studies from the Butaro Cancer Center in Rwanda and the Queen Elizabeth Central Hospital in Malawi, retrospective analyses of the TAT for pathology specimens showed a median TAT from specimen receipt to reporting of 32 days in Rwanda and 43 days in Malawi for specimens paid for out of pocket.^8,9^ In Malawi, a more prolonged median TAT of 101 days was reported for specimens that relied on state funds to pay for pathology services.^9^ In comparison, our study showed relatively shorter TAT in 2015, which was 6 and 7 days for biopsy and IHC specimens, respectively. Furthermore, our results showed a significant reduction in TAT for biopsy and IHC specimens analyzed at NHL and DML after 2012 compared with the prior period. These data attest to the positive effect of scale-up efforts for pathology services in Botswana. For IHC TAT, the data are also reflective of a trend in 2011 to 2012 when IHC was not routinely performed on all specimens compared with 2013 and after, when these were more routinely tested at either NHL or DML.

We identified the evaluation of surgical specimens as a critical area where no significant improvement in pathology TAT has been noted over the same time interval. The median TAT for surgical specimens in 2015 was approximately 2 months. Our analyses showed that the TAT from biopsy or surgery to receipt in the laboratory for specimens analyzed at PMH was 1 and 2 days for surgery and biopsy specimens, respectively, compared with a median TAT of up to 46 days for all other hospitals. There was not sufficient data per hospital to analyze the potential correlation between distance from NHL or DML and TAT for receipt in laboratory. More data will be needed to assess the effect of the location of peripheral health facilities on overall TAT.

On the basis of observation and review of current laboratory processes, we hypothesize that the delay in surgical pathology TAT is a result of preanalytical processes and less likely a result of delay in pathologists’ review. Surgical and biopsy specimens sent to pathology are grossed, microtomed, prepared on slides, and assigned to a pathologist for final review. For biopsy specimens, the histotechnologists perform all these preanalytical steps usually within 24 hours of receipt in the laboratory. In contrast, only a fraction of the daily volume of surgical specimens are grossed, sliced, and prepared on slides within 24 hours of receipt at the laboratory. This creates a daily backlog, which is compounded over time. This inefficiency results in a disproportionately prolonged preanalytical processing TAT for surgical specimens compared with biopsy specimens. This hypothesis is being tested by implementing a strict 24-hour preanalytical processing TAT for surgical specimens. Anecdotal data also suggest that biopsy specimens are assigned a higher priority by histotechnologists because it is felt that although biopsy specimens are urgent for establishing a diagnosis, surgical specimens are less important, suggesting that more education needs to be carried out to emphasize the prognostic and predictive significance of breast cancer surgical pathology. Future studies will measure the effect of these processing and educational interventions on TAT.

There are no specific guidelines regarding set targets for TAT in Botswana, and our goal is to achieve TATs that result in improvement in clinical outcomes in our patient population. The South African National Accreditation Society aims to achieve a TAT of 4 days for final reporting of breast biopsy specimens, 8 days for surgical specimens, and 14 days for IHC in the sub-Saharan African context. The College of American Pathologists requires a TAT of 2 days for 90% of routine cases to improve patient and treatment outcomes.^10^ Although our data suggest that strides have been made with TAT for biopsy and IHC specimens, and that these the median TATs are close to the targets within the sub-Saharan African context, there still remains significant work to be done with the processing and pathology review for breast surgical specimens. Surgical pathology provides critical prognostic information that helps guide decisions about adjuvant treatment of patients with breast cancer. A TAT of approximately 2 months has a costly clinical effect on patients. Efforts are currently under way by the MOHw, NHL, and University of Botswana, with support from ASCP, to improve efficiency in TAT through automation and standardization of pathology processes and education. Other interventions include the use of a slide scanner to upload slides to be remotely read by pathologists affiliated with the ASCP. The goal jointly set by pathologists at the NHL and Pathology Department at the University of Botswana at the Botswana Cancer Symposium in 2016 is to decrease pathology TAT for surgical specimens to 7 days by September 2017. Looking at the current human resources and available equipment, this is an achievable target.

The major strength of our study is that we analyzed more granular data on different types of specimens and TAT for both histology and IHC reporting. We were therefore able to assess different portions of breast pathology TAT that had improved and identify focused areas where additional interventions were needed to improve TAT and subsequently clinical care and outcomes for patients with breast cancer. For key stakeholders, the ability to analyze temporal trends as performed in our study provides necessary outcomes data needed for evaluating the effect of specific interventions. Some of the limitations of this study include the small sample size of the specimens reviewed. Despite the small sample size, our analysis showed a significant decline in TAT for biopsy and IHC reporting for the specified periods from 2011 to 2012 and 2013 to 2015. It is also possible that by including analysis from a private laboratory, the data may be skewed to reflect a lower TAT. Although only 25% of our samples were from DML, the analysis showed that in the private laboratory, TAT was consistently lower than that in the public sector. Last, our assessment of TAT did not include a quantitative assessment of time to receipt of pathology results by treating clinicians. Although this is beyond the scope of this report, we estimate that pathology results are available on the same day electronically when uploaded into the Botswana Integrated Patient Management System, which is an electronic medical record system available in all major cancer referral centers. For reports sent by mail to clinicians in remote health facilities via district hospitals, it usually takes 2 business days to reach the district health management team and up to a week or possibly longer to reach remote health care referring facilities. Consequently, pathology results sent by mail may lead to even longer TAT to clinicians than described in our study.

In conclusion, this study has shown a positive effect of pathology scale-up interventions, including the hiring of additional pathologists, the implementation of a pathology residency program, and the automation of histology processes as well as the availability of IHC at NHL and DML, on TAT for biopsy and IHC specimens. The local training of pathologists and the setting up of a bachelor’s degree in histotechnology and cytotechnology at the University of Botswana are laudable initiatives that will ensure the sustainability of an efficient and effective anatomic pathology service in the country. This will ultimately improve capacity in the country, create value-based pathology services, and affect sustainability of the discipline in the long term.
